# Female Physicians

**Published:** 1856-05

**Authors:** 


*Female Physicians.*—I have often wished, when reading the gross perversions of the truth which have been industriously brought before the public by interested persons, that the actual facts in regard to this subject might be so presented as to disabuse those who have been induced to credit the assertions so frequently and boldly made.

The proposition that women, as a sex, cannot practice medicine—that their weak physical organization renders them unfit for such duties and exposures —that their *physiological condition, during a portion of every month*, disqualifies them for such responsibilities — is too nearly self-evident to require argument. I therefore limit myself to a statement of the facts as regards midwifery alone, for the practice of which it has been especially claimed that they are competent.

It is asserted, in the first place, by the advocates of this claim, that were the habits of society less artificial, the process of child-bearing would be as easy and safe as in wild animals, calling for no intervention of science and skill. In the second place, they affirm, that in Europe the practice of midwifery is almost exclusively in the hands of females. Lastly, and as their weightiest argument, they declare that physicians are licentious, and that morality and delicacy require that they should be superseded.

But as respects savage nations, as well as in regard to domestic animals, we have abundant proof that no such immunity from pain and danger exists. On this point I beg leave to quote a small portion of the testimony lately collected by a distinguished English author,* who says: "A variety of recent valuable evidence (furnished chiefly in casual hints and allusions, the most unexceptionable kind of evidence) leads to a very different conclusion. So far is parturition from being *easy*, *expeditious and safe* in every instance, among barbarians, we have reason for thinking that *difficult* labors are as numerous with them as with us. In exemption from the usual causes of impeded labor, requiring the aid of science for the safe delivery of a patient, there is either no difference at all; or if there be, it will be found in the greater exemption, from such causes, of women in a state of civilization. Although much minute and specific information on this point is not to be expected, I have collected a number of remarks more or less bearing upon it. Long mentions, incidentally, the fact of a young woman of the Rat Nation being in labor a day and a night, without uttering a groan, the force of example acting so powerfully on her pride as not to allow her to express the pain she felt. A similar fact is stated in the voyage of Clarke and Lewis up the Missouri. Hearne, in his journal of an Expedition to the Northern Ocean, casually says, 'Here we were detained two days, owing to one of our women being taken in labor. She was not delivered till she had suffered for nearly fifty-two hours.' MacKenzie incidentally notices that on a particular time, the Indian hunter attached to the party returned, after a temporary absence, accompanied by his wife, leaving behind him his mother-in-law, in a helpless state, with three children, *and in labor with a fourth.* It came out that she had been left 'in a state of great danger.' Capt. Keating states respecting the Potawatomis, a tribe with which he associated for some time, and concerning whose manners his party gained much curious information, that labor was seldom fatal, but that many instances had occurred in which the child was so long *in being born*, that it was putrid when expelled. The same writer informs us that in answer to inquiries concerning the usual duration of labor in a tribe of Indians called Sauks, he was told that the pains of labor continued, in some instances, as long as four days. Among the Dacotas, the same party learned that parturition in some cases lasted from two to four days. We have another incidental notice of labor in an Indian in Franklin's Overland Journal. A Chippawyan woman fell in labor, in the woods, of her first child; and, on the third day after, died. In Krantz's account of the manners of the Greenlanders there occurs an allusion to parturition. Among others, those, it appears, are to find entrance to heaven who have died in childbirth. Messrs. Ellis and Bourne, who resided a great many years as missionaries in the South Sea Islands, have furnished me with valuable information concerning parturition as it occurs in those Islands.—Mr. Ellis says: 'Protracted and dangerous labors have generally been occa-

* Roberton.

sioned by mal-presentations.' Mr. Bourne says: 'The missionaries have saved many in difficult labors that would otherwise have died.' Long, the able historian of Jamaica, writes, in allusion to parturition among the slaves, that many children are annually destroyed, as well as their mothers, in the hands of the negro midwives. A writer in the Encyclopædia Brittanica has shown that for a long period midwifery has been practised in China by a set of men destined to the purpose by order of government. These men are called in whenever a woman has been above a certain number of hours in labor, and employ a mechanical contrivance for completing the delivery. The Chinese government, it is said, was led to make this provision in consequence of a representation that annually many women died undelivered, and that in a majority of cases the cause of obstruction might have been removed by simple mechanical expedients."

It is needless to add further evidence. We have seen that rude nations acknowlege the necessity for more or less assistance in the act of accouchement. This is further proved by the rude expedients resorted to by such nations to accomplish delivery. The circular fillet round the abdomen, tightened with great force by a dozen assistants, with the view of *forcing out the child;* the suspension of the woman by the heels, with the hope of altering the position of the infant, as practised among the Indians and Negroes, are examples of these.

As civilization advances, we find a far higher regard for all which concerns the welfare and safety of woman. It was this exalted regard which at last demanded the transfer of the responsibilities of the lying in chamber from the midwife to the educated accoucheur. The *results* of this change were, a diminution of the mortality incident to childbirth, in the course of half a century, *to half its former amount.* The *reasons* for it have been already partially alluded to; but one other of them is worth mentioning, as it furnishes a complete contradiction to the theories of the would-be reformers, who assert that women under such circumstances need more sympathy and gentleness than they receive from physicians of the other sex. This reason was, the *notorious harshness of the midwives.* With all the desire to display their importance and skill which belongs to half-cultivated minds, they sacrificed the comfort and even the safety of the patient to the endeavor to make a brilliant impression of their own ability. This is well known as regards those of Scotland and England at the present day. In regard to those of France, the writer was informed at Paris, that one reason why the midwives were not employed (except as a measure of economy by the poorer classes), was their extreme roughness, not to say cruelty, towards thier patients. The able author I have already cited says: "It is scarcely credible to what an extent they carry their interference in every stage of labor. It is no part of their system to trust to the unaided powers of Nature." Those who have had much opportunity to observe the harshness and neglect which many patients endure from their nurses, will be quite prepared to receive these statements as unexaggerated.

But we are told, in the second place, that in Europe, and especially in France and Germany, the practice of midwifery is almost the exclusive province of females. I submit the following facts, obtained by personal observation. The government does all in its power to render the "sage femmes," or midwives, as far as they can be, competent, by providing for them a system of instruction under the direction of the faculty of medicine, and by requiring them to pass two distinct examinations before they are permitted to practice. But, even after such qualification (far superior to anything dreamed of in this country), they have been found so unskillful that they are forbidden, by law, to continue in charge of a difficult case, or to apply instruments, without calling in a physician. Even the eminent midvives who have the superintendence of the Maternité and the other large lying-in hospital at Paris, with their experience of thousands of cases, do not have the responsibility of the management of difficult labors. The physician who has charge of the hospital, or if he cannot be found, his substitute, is sent for. If neither of them can be found, notice is left at their houses; but, if delay be inadmissible, the house physician, not the chief midwife, takes charge of the case. Educated as we have seen, the midwives enter upon the discharge of the duties of practice, but not to be welcomed and patronized by the delicate and refined portion of their sex. In Paris, some find employment among the lower classes; others sustain themselves by keeping houses for accouchement, of which the signs may be noticed in all the less respectable quarters of the city. These houses afford a cheap resource for the wives of such small tradesmen as find their apartments at home too limited for their comfort during confinement, as well as for a large class who desire secrecy. Here the young girl, not a wife, becomes a mother; and the widow hides the consequences of her "indiscretion." Hence, perhaps, the child is sent to the basket of the Foundling Hospital, very probably to fall a victim to its want of maternal care; and the mother, having paid her forty francs for the accouchement and the nine davs allotted her, returns to her position in society. In the rural districts, as in Great Britain, some midwives obtain a partial support in the small hamlets which are too far from large places to allow of the services of a physician being readily procured for such occasions.

Many of equivocal reputation occupy the ranks of the midwives, who having pursued an improvident career as grisettes, find themselves, at middle age, with no resource so convenient as the vocation of the sage femme. That such persons should be unscrupulous in practicing the illegitimate arts of their calling, as well as its honorable duties, need surprise no one. In Great Britain the education of midwives is less methodical; but they are similarly sustained, by the lower classes only, not as a matter of choice, but of economy. The competence of some may be judged of from a case lately brought before the London courts, where a midwife (who had been a pupil at a London lying in hospital), after the patient had been delivered, dragged the womb itself out of the body, and then, supposing that this organ was something which ought to be removed, *tore it away from the woman*, causing her speedy death.

If, then, midwives have still a recognized existence in European countries, they do not owe it to any superior delicacy or higher morality; but, as I have shown, to circumstances inseparable from a poor or sparsely scattered population. These circumstances do not exist in even the most thinly-settled portions of New England, and the more valuable services of the physician have been within the reach of all, no matter how poor or how distant.

But the public have been told, *not by ladies*, but by men whose grossly indelicate works do not go to prove *them* the fittest judges, that the confidence of the sex is abused by physicians, and that to employ them is an offence against the higher sentiments of woman's nature. Every pure-minded lady denies the libel, as regards her trusted medical adviser and the profession at large, as well as herself. Incapable of the indelicacy of thinking and acting as if, in any matters concerning the health of herself and her children, there could be any question of sex, she describes to her physician, without hesitation or reserve, the physiological or pathological phenomena in regard to which she solicits his advice; knowing that he receives her confidence in the same spirit. There may be exceptions in morality among physicians; but where can an equal number be found, in any class of society, whose conduct is *as irreproachable.* No objection is made to the admission of clergymen to intimate and confidential relations with the other sex, although these relations take place under circumstances infinitely more likely to lead into temptation, and though the community has witnessed more instances of exposure of misconduct on the part of the clergical than of the medical profession.

I trust I have fully proved, that so far from being a benefit to society, so far from enhancing the purity and delicacy of female character, it would be a misfortune to both that any retrograde step should be taken, as regards the qualifications and character of the medical attendant. The duties of the accoucheur are not limited to the service rendered on a single occasion. He must see that mother and child are doing well, and take every precaution to avert any germ of future disease. Is he, with his intimate knowledge of the whole constitution, his skill acquired by years of thought and culture, any too competent for these important responsibilities?

Some wise and worthy men have been anxious that the experiment should be tried; some clergymen have been persuaded to give an opinion on a question of which they are most unqualified judges; but the public have given but a chilling support to the languishing experiments which they have been forced to witness in the sham education of females. Nor will the occurrence, within a few weeks of each other, and within a short distance of Boston, of two cases, where the gross ignorance of two of the professedly educated females, cost the life of one patient, and made another the subject of infirmity which renders life a burden, be likely to exalt the plan in public favor. But like its coëval Bloomerism, the scheme has already received its deserts. It contains within itself the elements of failure; for, as one of its advocates remarked, "the girls do n't like to dissect." They did not seem to like, either, to devote more than three months to a course of medical education.

I have offered these too long remarks, Messrs. Editors, although you have so ably disposed of part of the question in your No. of the 1st November last, in hope that the statement of facts may enable some in the profession to refute the assertions which have been so freely made, and to give a satisfactory answer to the appeal which is now and then made to them for the truth in regard to the merits of this question.—*Boston Med. Journal.*

